# Everter-Assisted Arterial Coupling in Abdominal-Based Breast Reconstruction: A Preclinical Study and Retrospective Feasibility Case Series

**DOI:** 10.3390/cancers18203343

**Published:** 2026-10-16

**Authors:** Amir Dehdashtian, Michael Sorkin, Paul S. Cederna, Adeyiza O. Momoh

**Affiliations:** 1 Section of Plastic Surgery, Department of Surgery, University of Michigan Medical School, Ann Arbor, MI 48109, USA; 2 Department of Plastic and Reconstructive Surgery, The Ohio State University Wexner Medical Center, Columbus, OH 43210, USA

**Keywords:** arterial coupling, microvascular anastomosis, abdominal-based flap, breast reconstruction, everter device, internal mammary artery, microsurgery, free tissue transfer

## Abstract

**Background/Objectives:** Arterial coupling in microsurgery remains technically challenging despite widespread venous coupler adoption. We developed a novel everter device to facilitate arterial anastomosis using mechanical couplers and evaluated its feasibility and potential efficiency in abdominal-based breast reconstruction. **Methods:** Five pigs underwent paired superior epigastric artery flap reconstruction with everter-assisted and hand-sewn arterial anastomoses, analyzed using a paired *t*-test. A retrospective clinical series included 12 patients, 22 reconstructed breasts, 14 coupling attempts, and 8 primary hand-sewn recipient arterial anastomoses. The primary clinical outcome was successful coupling without revision or conversion. Clinical timing comparisons were descriptive. **Results:** All five porcine arterial couplings were successful. Mean arterial time was 8.47 ± 2.83 min versus 20.26 ± 3.32 min for hand-sewn controls, a paired reduction of 11.79 min (95% CI, 5.86 to 17.72; *p* = 0.005). Clinical coupling succeeded in 9 of 14 attempts (64.3%). Five attempts required conversion (35.7%), including three thrombotic and two technical failures. All were converted intraoperatively, with no flap loss. Mean arterial times were 9.2 ± 4.2 min for successful coupling (n = 8) and 20.2 ± 8.0 min for primary hand-sewn anastomosis (n = 6), an approximately 55% difference. **Conclusions:** Everter-assisted arterial coupling demonstrated clinical feasibility and shorter mean arterial times when successfully completed. These findings support further refinement and evaluation of the technique to establish safety and overall procedural efficiency.

## Simple Summary

Autologous free tissue transfer is a preferred method of breast reconstruction following mastectomy and depends on microvascular anastomosis of the vessels perfusing the transferred flap. Mechanical coupling devices have become standard for venous anastomosis, yet arterial anastomosis continues to rely on hand suturing, as the thicker and more elastic arterial wall resists eversion onto the coupler pins. This reliance increases anastomotic complexity and prolongs operative and ischemia times. The present study evaluated an everter device that facilitates engagement of the arterial wall onto the coupler pins in a porcine model and an initial clinical series. The clinical series included 12 patients and 22 reconstructed breasts, with 14 attempted couplings. Nine attempts were successful (64.3%); five required conversion to hand-sewn anastomosis (35.7%), including three thrombotic failures. No flap loss was observed. Clinical timing comparisons were descriptive, and the effects of technique and surgeon could not be separated. These early findings support further evaluation of technical feasibility, safety, and overall procedural efficiency.

## 1. Introduction

Autologous breast reconstruction using free tissue transfer has become the preferred method for many patients seeking post-mastectomy reconstruction as it offers long-term satisfaction compared with implant-based approaches [1,2,3]. Among the various donor sites available, the deep inferior epigastric perforator (DIEP) flap has emerged as a workhorse option, providing adequate tissue volume while preserving abdominal wall integrity and minimizing donor-site morbidity [4,5,6]. Central to the success of any free tissue transfer is the reliability and efficiency of microvascular anastomoses, which must provide immediate patency, maintain long-term integrity, and ideally be performed with technical ease and time efficiency [7,8].

The current concept of mechanical vascular coupling dates back to 1960, when Gordon P. Holt, a medical student at Northwestern University working under the mentorship of Thomas Starzl and F. John Lewis, developed a device with interdigitating holes and pins specifically designed for vessels 4 mm or larger [9]. Nakayama and colleagues subsequently refined this concept in 1962, introducing a coupler consisting of two metal rings with twelve interlocking pins and corresponding holes, and later work by Ostrup and Berggren in 1986 further optimized the device into the predecessor of contemporary commercial systems [9,10,11]. Importantly, these devices were not originally designed exclusively for venous anastomoses; rather, their clinical use became predominantly venous because of practical limitations encountered with arterial coupling [9,12].

Venous coupling has since achieved widespread acceptance in microsurgery, with high patency rates, reduced anastomotic time, and complication profiles comparable to or better than hand-sewn techniques [13,14,15]. In contrast, arterial coupling has remained technically challenging and far less commonly adopted. The primary obstacles relate to the thicker, more muscular and elastic arterial wall, which resists eversion onto coupler pins and often results in intimal injury during manipulation [16,17,18]. These factors increase the risk of luminal compromise and thrombosis, particularly in smaller-diameter vessels [19].

**Figure 1:**
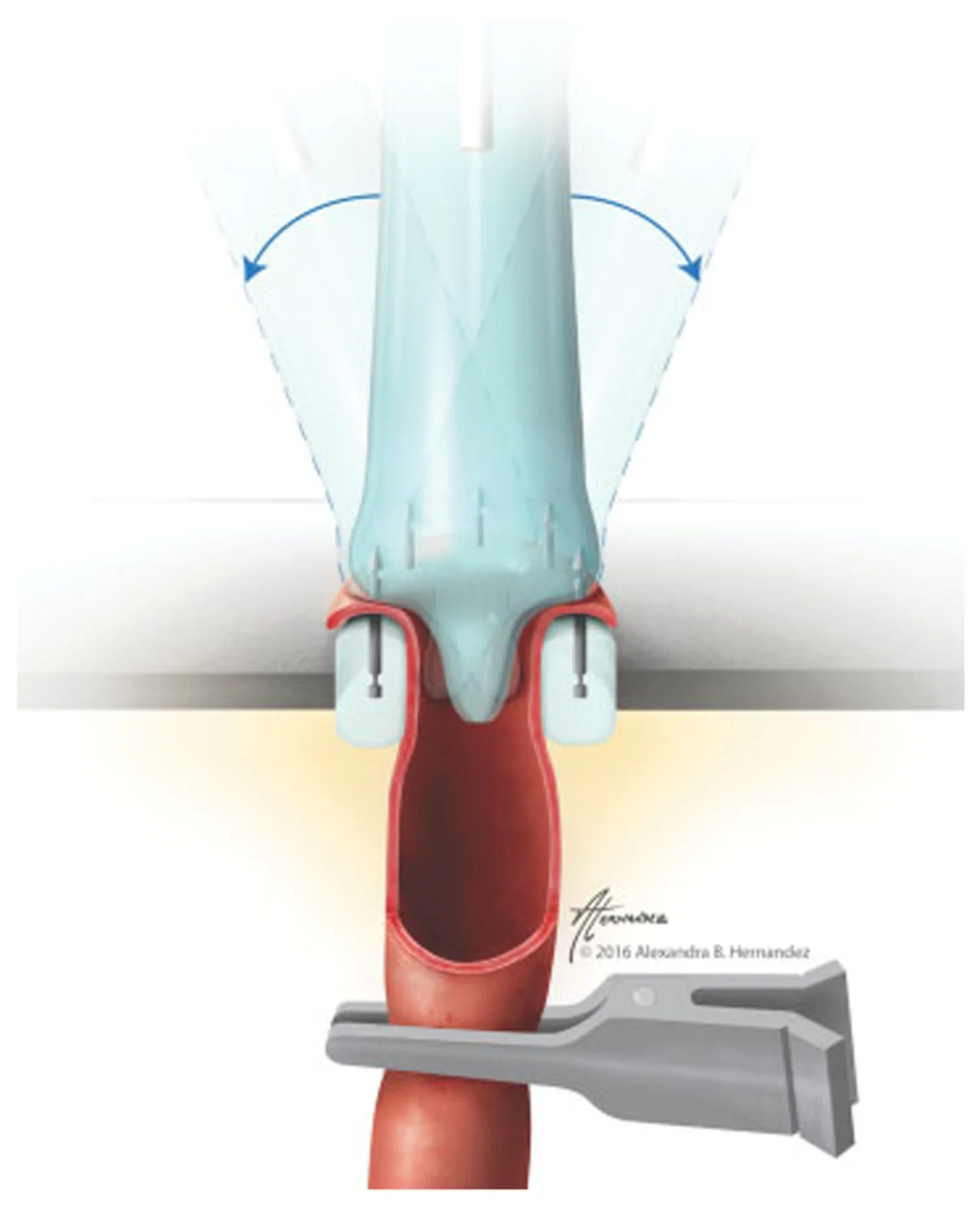
Schematic of the arterial everter device and its mechanism of action. The device is a pen-like, tapered medical-grade silicone cone, rigid proximally for precise control and soft distally to minimize vessel trauma. After each arterial end is passed through the microvascular coupler ring with sufficient redundancy, the tapered silicone tip is introduced into the arterial lumen and advanced against the ring. The compressible tip allows the coupler pins to pierce both the arterial wall and the device simultaneously, producing circumferential, controlled eversion of the vessel wall with simultaneous pin engagement in a single coordinated motion. This standardizes eversion, distributes compressive force evenly around the vessel, and overcomes the natural recoil of the thick arterial wall without repeated forceps manipulation. (Adapted from Sando et al. [16]).

To address these limitations, our group developed a novel arterial everter device designed to facilitate safe and efficient arterial coupling [16]. The device consists of a pen-like, tapered medical-grade silicone cone that is rigid proximally for precise control and is soft distally to minimize vessel trauma. When inserted into the arterial lumen and advanced over the coupler pins, the compressible silicone tip allows the pins to pierce both the arterial wall and the device simultaneously, achieving circumferential, controlled eversion of the vessel wall with simultaneous pin engagement in a single coordinated motion. This design aims to standardize eversion, reduce focal trauma from repeated forceps manipulation, and overcome the natural recoil of thick arterial walls (Figure 1). Preclinical validation in a porcine femoral artery model demonstrated the feasibility and safety of this approach with 100% immediate patency and reduced anastomotic time by 74% compared to hand-sewn technique [16].

In theory, extending the use of coupling devices from venous to arterial anastomoses offers several potential advantages. These include a standardized and reproducible technique that reduces operator variability, rigid stenting of the anastomotic site that may protect against kinking and vasospasm, elimination of intraluminal suture material that could act as a nidus for thrombosis, and consistent intima-to-intima apposition that may promote optimal healing [13,20]. In the context of complex microsurgical breast reconstruction, particularly bilateral DIEP flap procedures, any reduction in anastomotic time and technical complexity may translate into shorter ischemia times, decreased overall operative duration, and increased availability of DIEP flap breast reconstruction.

The present study evaluated everter-assisted arterial coupling in a porcine superior epigastric artery (SEA) free-flap model [21] and an initial retrospective case series of abdominal-based breast reconstruction. The clinical objective was to describe feasibility and technical limitations through the initial experience, with successful coupling without revision or conversion as the primary outcome. Secondary outcomes included arterial anastomosis time, ischemia time, flap survival, and complications.

## 2. Materials and Methods

This study combined an exploratory preclinical porcine SEA free-flap experiment [21] with an early retrospective feasibility case series of abdominal-based breast reconstruction at a single academic medical center. The animal study was conducted between December 2019 and March 2020. The clinical series comprised 11 patients treated between December 2019 and March 2020 and one additional patient treated in March 2026. The animal protocol was approved by the Institutional Animal Care and Use Committee (IACUC), and the clinical records were reviewed under Institutional Review Board (IRB) approval for retrospective research.

### 2.1. Preclinical Animal Study

The preclinical component was an exploratory feasibility study involving five male Yorkshire swine (43.6 ± 3.3 kg), each serving as its own control. Animals were obtained through the University of Michigan Unit for Laboratory Animal Medicine (ULAM) and acclimated for 5–7 days with positive-reinforcement training to minimize stress and facilitate handling.

**Figure 2:**
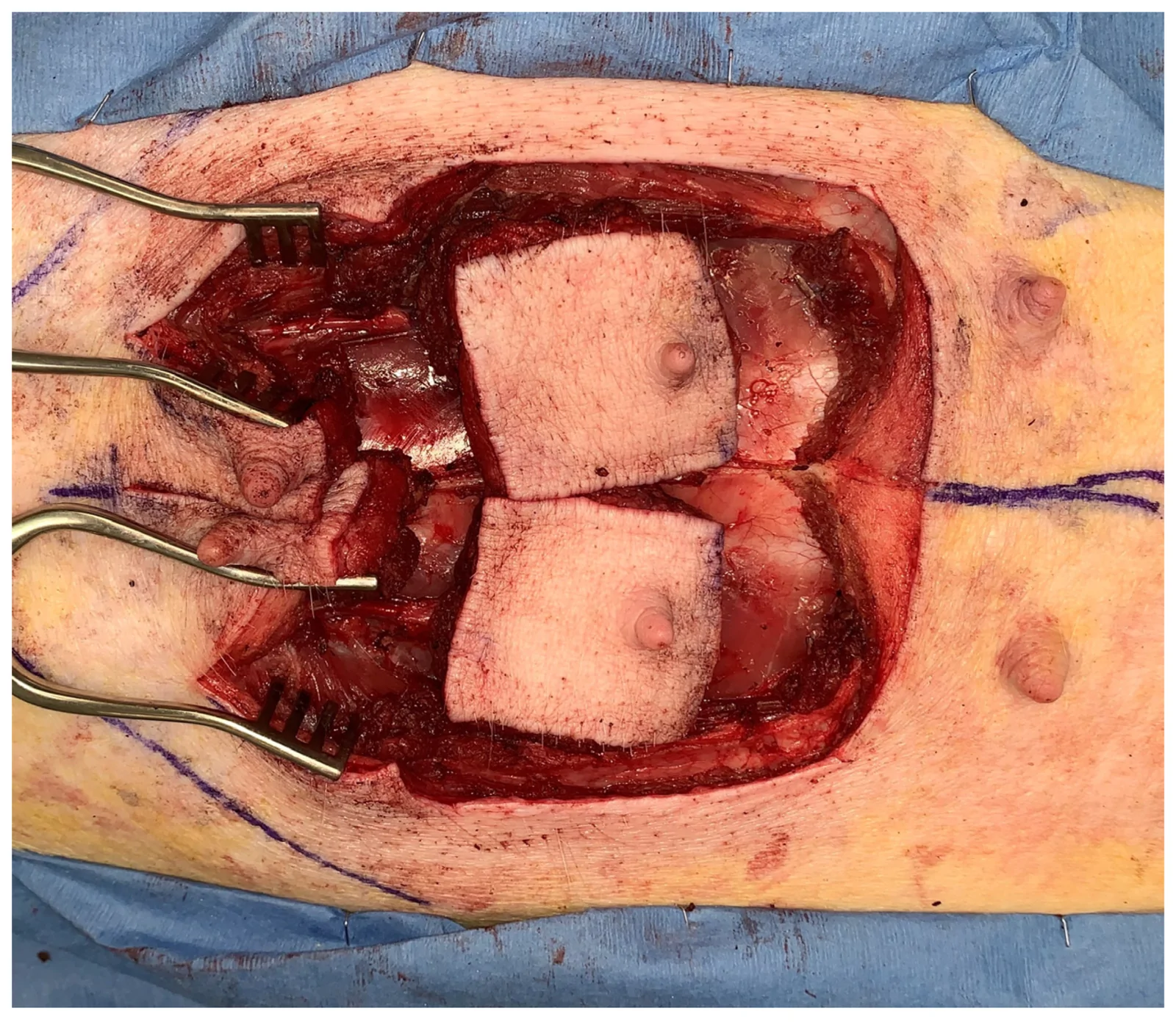
Bilateral superior epigastric artery (SEA) flaps raised in the porcine preclinical model. Two flaps are elevated on the ventral abdomen, each perfused by its SEA pedicle, providing paired anastomotic sites for evaluation of everter-assisted arterial coupling against hand-sewn control within the same animal.

Animals were sedated with ketamine/xylazine, maintained under inhaled isoflurane anesthesia, and positioned supine on a warming blanket with continuous monitoring. Surgery was performed aseptically. Each animal underwent bilateral SEA flap elevation and transfer to the contralateral superior epigastric recipient vessels (Figure 2). The side receiving everter-assisted arterial coupling was randomly assigned, and the contralateral artery was anastomosed with interrupted hand-sewn 8-0 Nylon sutures. Venous anastomoses on both sides used standard couplers (GEM system, Synovis Micro Companies Alliance, Inc., Birmingham, AL, USA). Arterial completion times were recorded using a timer. Animals were monitored for 7 days with flap viability assessment and were then euthanized per protocol. Postoperative assessments were performed by one of the authors who was not blinded to technique. The approved protocol specified perioperative carprofen, additional buprenorphine as needed, assessment of Doppler flow, flap color and capillary refill, and veterinary consultation for severe adverse findings.

### 2.2. Clinical Study

This retrospective case series included 12 patients undergoing abdominal-based breast reconstruction with attempted everter-assisted recipient arterial coupling. Ten patients had bilateral and two had unilateral reconstruction, yielding 22 flaps, one per reconstructed breast. Coupling was attempted bilaterally in two patients, unilaterally in eight bilateral reconstructions, and in both unilateral reconstructions, giving 14 coupling attempts and 8 primary hand-sewn recipient arterial anastomoses. The cohort included two muscle-sparing transverse rectus abdominis myocutaneous (MS-TRAM) flaps and two stacked hemi-abdominal extended perforator (SHAEP) flaps in one bilateral reconstruction. Each SHAEP flap comprised DIEP and superficial circumflex iliac artery perforator (SCIP) components connected by an additional hand-sewn arterial anastomosis and coupled venous anastomosis, separate from the recipient IMA–DIEA anastomosis.

**Figure 3:**
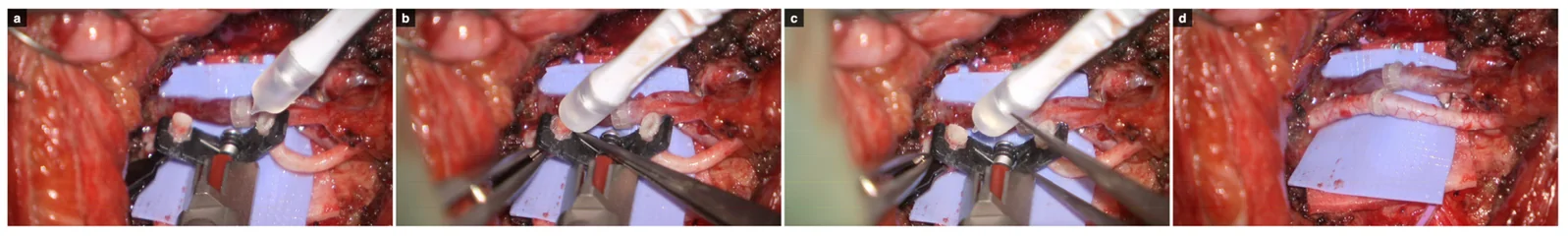
Intraoperative application of the everter device for coupling of the deep inferior epigastric artery (DIEA) to the internal mammary artery (IMA) during DIEP flap breast reconstruction.

Abdominal-based flaps were harvested using standard techniques following preoperative markings to optimize volume and perfusion. All patients had preoperative CT angiography to evaluate the perforators. The internal mammary vessels were exposed through the anterior intercostal space via resection of the third or fourth costal cartilage. Venous anastomoses were performed using standard venous couplers based on vessel size. Arterial anastomoses were attempted using the everter device (Figure 3) by the senior author (AOM). Arterial coupler size was selected using an intraoperative vessel sizer (GEM microvascular anastomotic coupler, Synovis Micro Companies Alliance/Baxter, Deerfield, IL, USA). Each arterial end (DIEA and IMA) was passed through its coupler ring, leaving enough redundancy to fold the vessel wall back over the pins. The tapered silicone tip of the everter was then introduced into the arterial lumen and advanced against the ring, everting the wall and driving the coupler pins through its full circumference in a single motion. Because the soft tip allows the pins to pierce both the arterial wall and the device itself, compressive force is distributed evenly around the vessel, and the wall is secured without repeated forceps manipulation [16]. Any pin that failed to engage after a single pass was seated with angled forceps, and once both vessel ends were secured on the coupler rings, the rings were approximated to establish arterial continuity. In cases where the everter device was unsuccessful in achieving adequate pin engagement, conversion to standard hand-sewn technique was performed. Anastomoses were performed sequentially within a co-surgeon model [22]. The primary surgeon performed all coupling attempts, whereas the co-surgeon performed primary hand-sewn anastomoses. Side assignment and operative order depended on surgeon availability and workflow; coupling was not consistently performed first or second.

Successful coupling was defined as a patent recipient arterial anastomosis at completion of surgery without revision or conversion; all attempts were retained in the denominator. Technical failure indicated inability to complete coupling because stable arterial eversion and pin engagement could not be achieved. Thrombotic failure indicated thrombosis at the coupler after initial restoration of flow. Arterial timing used the same definition for coupled and primary hand-sewn anastomoses: from placement of the microscope in the operative field and commencement of vessel preparation to clamp release with restoration of flow. SHAEP intraflap connections were excluded from this measurement. Ischemia time was defined as the interval from clipping the abdominal flap pedicles to clamp release after completion of the recipient anastomoses in the chest. Clinical data and outcomes were extracted retrospectively from medical records, operative reports, and nursing documentation. Complications were recorded descriptively by type and treatment, with early events defined as occurring during the index admission and late events after discharge.

### 2.3. Statistical Analysis

Continuous variables are presented as mean ± standard deviation with available sample sizes; categorical variables are reported as counts and percentages. Each summary included only observations with recorded values; missing values were not imputed. Preclinical arterial times were compared using a two-sided paired *t*-test, with each animal as the paired unit; the mean paired difference and its 95% confidence interval were calculated from the five pairs. Normality of the within-animal time differences was assessed using visual inspection of a normal Q-Q plot. Clinical arterial anastomosis and flap ischemia times were summarized descriptively; no hypothesis tests were performed for these outcomes. Exploratory 95% confidence intervals for mean within-patient timing differences were calculated from complete pairs using the *t* distribution. The two SHAEP flaps were excluded from ischemia-time analysis because additional intraflap anastomoses were performed after abdominal pedicle clipping and before the recipient IMA–DIEA anastomosis. Coupling success is reported with a 95% Wilson confidence interval, which does not account for patient clustering.

## 3. Results

### 3.1. Preclinical Porcine Study

All arterial anastomoses were completed successfully in both preclinical groups, with no conversions, device malfunctions, or technical failures. All remained patent through postoperative day 7, with no thrombotic events or flap loss observed. Mean arterial time was 8.47 ± 2.83 min with everter assistance and 20.26 ± 3.32 min with hand-sewn anastomosis. The mean paired reduction was 11.79 min (95% CI, 5.86 to 17.72; paired *t*-test, *p* = 0.005), corresponding to a 58.2% reduction in group mean time (Table 1). Venous couplers were 2.0 mm in seven anastomoses and 2.5 mm in three. All arterial couplers were 2.0 mm. In one animal, a unilateral fishmouth technique was used on the donor SEA to allow use of a 2.0 mm coupler. No intraoperative or postoperative complications were reported.

### 3.2. Clinical Study

#### 3.2.1. Patient Demographics and Clinical Characteristics

Twelve patients underwent reconstruction of 22 breasts, with 14 attempted recipient arterial couplings (Table 2; Supplementary Figure S1). Mean age was 44.8 ± 3.9 years and mean body mass index was 31.6 ± 7.5 kg/m^2^. Ten out of twelve patients were never-smokers, and two were former smokers. Three of 12 had prior radiation therapy, 7 of 12 underwent immediate reconstruction, and 10 of 12 underwent bilateral reconstruction. Mean follow-up duration was 19.5 ± 17.1 months (Table 2).

#### 3.2.2. Arterial Coupler Success and Operative Outcomes

**Figure 4:**
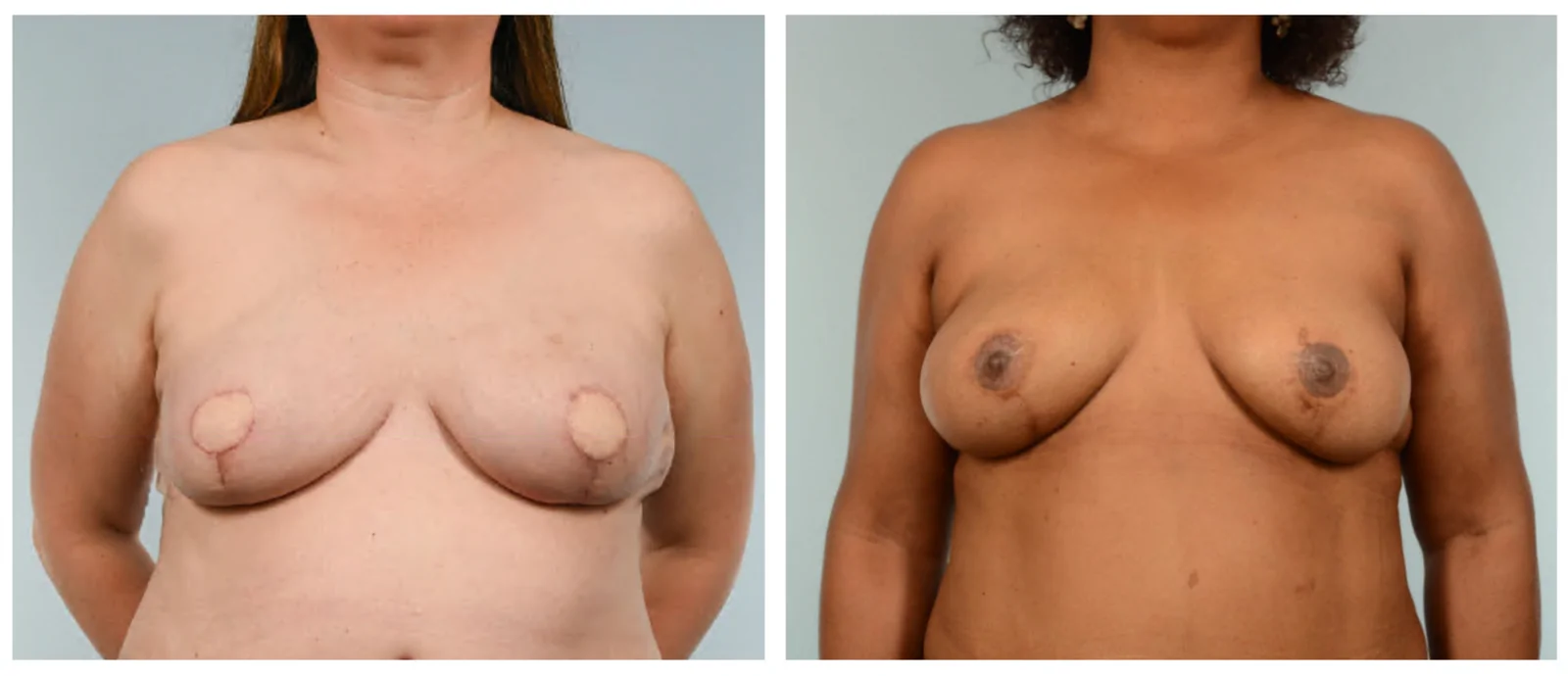
Representative postoperative results in patients who underwent everter-assisted arterial coupling. (**Right panel**): a patient with a successful everter-assisted coupled arterial anastomosis on the right side and a hand-sewn arterial anastomosis on the left. (**Left panel**): a patient with a failed everter-assisted attempt on the left side that was converted intraoperatively to a hand-sewn anastomosis, with a hand-sewn arterial anastomosis on the right.

Of 14 attempted recipient arterial couplings, 9 were successful (64.3%; 95% CI, 38.8% to 83.7%). Five required conversion (35.7%), including three thrombotic failures (21.4%) and two technical failures (14.3%). All were converted intraoperatively to hand-sewn arterial anastomosis; no flap loss was observed (Figure 4). In all three thrombotic failures, flow was initially established but became compromised before flap inset. Exploration identified thrombosis at the coupler. The involved segment was excised, the lumen was flushed, and a hand-sewn anastomosis was performed without attempted revision of the coupled anastomosis. The two technical attempts were abandoned because arterial recoil prevented stable eversion and engagement on the pins; neither coupler was closed, and the clamps were not released before conversion. The nine successful couplings occurred in seven patients, including both patients with bilateral attempts. Thirteen attempted arterial couplers were 2.0 mm, and one was 2.5 mm. Mean flap weight was 696.2 ± 351.4 g (n = 13). Mean recorded venous time was 3.9 ± 2.2 min (n = 12 attempted sides), ischemia time was 59.3 ± 17.5 min (n = 12 attempted sides, excluding the two SHAEP flaps), and hospital stay was 3.5 ± 0.5 days.

One patient had an immediate intraoperative complication involving loss of venous signal identified during flap inset and underwent a second venous anastomosis. Early postoperative complications occurred in 2 of 12 patients (16.7%), both involving breast hematomas requiring washout. Recorded bleeding sources were likely the pectoralis muscle in one and a transected lateral perforator in the other. Nine late events occurred in 7 of 12 patients (58.3%): fat necrosis in three, wound dehiscence in three (two abdominal and one breast), abdominal drain-site cellulitis in one, and delayed hematoma in two. Patients 1 and 9 each had two late events; the other five affected patients each had one. The breast wound dehiscence occurred on the primary hand-sewn side. Treatments included dressing changes, antibiotics, drainage, and liposuction for fat necrosis. No flap loss was observed. Patient and operative factors by coupling outcome are summarized in Table 3; individual clinical characteristics, failure details, and postoperative complications are provided in Table S1.

#### 3.2.3. Arterial Anastomosis Time

Among three complete within-patient pairs, the mean arterial time difference (hand-sewn minus coupled) was 8.5 min (95% CI, −17.4 to 34.3). One pair favored hand-sewn anastomosis. As a secondary descriptive summary, mean time was 9.2 ± 4.2 min for successful coupling (n = 8), compared with 20.2 ± 8.0 min for primary hand-sewn anastomosis (n = 6). Individual within-patient comparisons are provided in Table S2.

In the three cases subsequently complicated by thrombosis, initial coupling took 10.4 ± 6.1 min. These measurements excluded subsequent conversion to hand-sewn anastomosis, which was not timed separately.

#### 3.2.4. Flap Ischemia Time

Successful arterial coupling was also associated with shorter flap ischemia time. Mean ischemia time was 50.9 ± 10.7 min for successful coupling (n = 7 excluding SHAEP flaps), compared with 77.5 ± 22.0 min for primary hand-sewn anastomosis (n = 8), an observed difference of approximately 27 min. Flaps requiring conversion had a mean ischemia time of 71.2 ± 19.2 min (n = 5). Among five complete within-patient pairs, the mean ischemia time difference (hand-sewn minus coupled) was 32.2 min (95% CI, −1.0 to 65.4). Individual paired comparisons are provided in Table S2.

## 4. Discussion

This study extends the preclinical evaluation of the everter device to clinical arterial coupling in abdominal-based breast reconstruction, addressing the technical challenge of arterial-wall eversion onto coupler pins. Successful coupling in 9 of 14 attempts using the internal mammary artery as the recipient demonstrates the potential for this approach in clinical microsurgery. When successful, coupling was associated with shorter mean arterial anastomosis times and a 27 min shorter flap ischemic interval compared with primary hand-sewn anastomosis. These findings provide an encouraging basis for further evaluation of the device as a means of simplifying arterial anastomosis and improving operative efficiency. The five conversions, including three thrombotic failures, identify technical limitations that warrant refinement. All were managed intraoperatively without flap loss, underscoring the importance of promptly recognizing unsuccessful coupling and maintaining readiness for hand-sewn conversion.

The paired porcine experiment demonstrated consistent technical performance, with successful coupling in all five animals and a 58.2% reduction in arterial anastomosis time. These results extend earlier preclinical evaluation of the everter device [16] and complement experimental investigation of arterial coupling in other microsurgical settings [23]. The clinical series provides the next step in that translation: coupling could be completed using the internal mammary artery, while the unsuccessful attempts revealed the difficulties encountered beyond a controlled experimental setting.

The clinical findings should be considered in the context of the recipient vessels and reconstructive procedures represented in previous reports. Pafitanis et al. reported a 92.1% success rate across 670 arterial anastomoses, of which 117 were in breast reconstruction [19]. In a series of 80 breast reconstructions, Spector et al. successfully coupled 60 of 69 flaps based on the deep inferior epigastric artery (87%); coupling was used with thoracodorsal recipients but not with internal mammary recipients [24]. Rad et al. demonstrated coupling to internal mammary intercostal perforators in nine cases [25]. These studies establish the potential of arterial coupling while illustrating that recipient selection is central to its application. Our series adds experience with everter-assisted coupling to the internal mammary artery itself.

Arterial-wall characteristics provide a rationale for the everter design. Lohasammakul et al. described differences in elastic-fiber content between internal mammary and thoracodorsal arteries and identified the thoracodorsal artery as more favorable for coupling [26]. Arterial recoil can make stable eversion and circumferential pin engagement difficult, which is the technical problem the everter is designed to address. In a more recent series of 105 patients, Kim et al. used intraoperative criteria of absent atherosclerosis, intact intima, and adequate vessel laxity to guide arterial coupler use [27]. These observations support further evaluation of vessel assessment alongside device refinement, rather than assuming that the same coupling approach will be suitable for every artery.

The comparison of successful and unsuccessful attempts is hypothesis-generating. Converted flaps were numerically heavier than successfully coupled flaps (866.0 ± 331.6 versus 590.1 ± 339.6 g), while the difference in patient BMI was modest (32.9 versus 30.6 kg/m^2^). The two highest-BMI patients underwent successful coupling. Although obesity and elevated blood pressure have been associated with arterial stiffness [28,29], neither BMI nor flap weight directly describes the artery being anastomosed. These findings favor investigating vessel-level characteristics over proposing a selection threshold based on body habitus from this small series.

The unsuccessful attempts identify two distinct priorities for technical refinement. In the two technical failures, recoil prevented stable eversion and pin engagement, prompting conversion before coupler closure or clamp release. The three thrombotic failures followed initial restoration of flow and were recognized before flap inset. This distinction emphasizes that secure pin engagement and initial patency are separate checkpoints. Intraoperative conversion preserved all flaps, but thrombosis required segment excision and further anastomosis. These observations support close assessment of flow before inset and a low threshold for conversion, and identify intraoperative vessel assessment as a priority for future evaluation.

This study is limited by its small sample and retrospective, single-institution clinical design. All coupling attempts were performed by one experienced microsurgeon, whereas primary hand-sewn anastomoses were performed by a co-surgeon; technique and operator effects therefore cannot be separated. Side assignment and operative order followed workflow, and bilateral observations were not independent. Clinical comparisons were consequently presented descriptively. The larger difference in ischemia time than in arterial anastomosis time may also reflect operative sequence and flap complexity, even after excluding SHAEP reconstructions [30]. Unrecorded hand-sewn rescue durations prevent estimation of the overall time required for an attempted-coupling strategy. The inventor-surgeon did not participate in data extraction or analysis, although his operative role remains a potential source of bias. The animal sample was small and postoperative assessments were unblinded. No flap loss was observed, but this initial experience cannot establish a comprehensive comparative safety or predict performance across surgeons.

The observed time advantage with successful coupling and the specific failure patterns provide a practical basis for the next stage of evaluation. Prospective studies should examine vessel characteristics, reproducibility across surgeons, and complete time to definitive patency, including conversion when required. These measures would determine whether refinements that improve coupling success translate into a consistent procedural benefit while maintaining reliable flap perfusion.

## 5. Conclusions

Everter-assisted arterial coupling was feasible in this initial clinical series, with successful coupling in 9 of 14 attempts and shorter mean arterial anastomosis times when successful. Five attempts required intraoperative hand-sewn conversion, with no flap loss observed. These findings support continued refinement of the technique and prospective evaluation of its safety, reproducibility, and overall procedural efficiency.

## Figures and Tables

**Table 1:** Arterial anastomosis times in five paired porcine experiments. The confidence interval and two-sided *p*-value are based on within-animal differences.

Parameter	Hand-Sewn Control	Everter-Assisted	Mean Difference (95% CI)	*p*-Value
Mean ± SD (minutes)	20.26 ± 3.32	8.47 ± 2.83	11.79 (5.86–17.72)	0.005
Range (minutes)	16.33–24.40	5.63–12.27		

**Table 2:** Patient and clinical characteristics.

Characteristic	Value
Age (years), mean ± SD (range)	44.8 ± 3.9 (37–51)
Body mass index (kg/m^2^), mean ± SD (range)	31.6 ± 7.5 (21.2–46.2)
Never-smoker, n (%)	10 (83.3)
Prior radiation therapy, n (%)	3 (25.0)
Hypertension, n (%)	3 (25.0)
Diabetes/prediabetes, n (%)	1 (8.3)
Immediate reconstruction, n (%)	7 (58.3)
Bilateral procedures, n (%)	10 (83.3)
Follow-up (months), mean ± SD (range)	19.5 ± 17.1 (1.3–55.5)

**Table 3:** Patient and operative factors by arterial coupler outcome.

Variable	Successful (n = 7 Patients)	Failed (n = 5 Patients)
Demographics		
Age (years), mean ± SD	43.7 ± 3.8	46.2 ± 4.1
BMI (kg/m^2^), mean ± SD	30.6 ± 8.9	32.9 ± 5.6
Comorbidities		
Hypertension, n (%)	1 (14)	2 (40)
Diabetes/prediabetes, n (%)	0 (0)	1 (20)
Prior radiation therapy, n (%)	2 (29)	1 (20)
Operative characteristics		
Flap weight (grams), mean ± SD	590.1 ± 339.6	866.0 ± 331.6
Ischemia time (minutes), mean ± SD	50.9 ± 10.7	71.2 ± 19.2
Hospital stay (days), mean ± SD	3.6 ± 0.5	3.4 ± 0.5
Reconstruction Characteristics		
Immediate reconstruction, n (%)	3 (43)	4 (80)

Values are reported per patient except flap weight (n = 8 successful and 5 failed flaps) and ischemia time (n = 7 successful and 5 failed flaps).

## Data Availability

The data presented in this study are available on request from the corresponding author. The data are not publicly available owing to patient privacy restrictions.

## Supplementary Materials

The following supporting information can be downloaded at: https://www.mdpi.com/article/10.3390/cancers18203343/s1, Figure S1: Clinical outcomes of everter-assisted arterial coupling; Table S1: Clinical characteristics, arterial coupling outcomes, and complications; Table S2: Complete within-patient comparisons of arterial anastomosis and flap ischemia times.

## Abbreviations

The following abbreviations are used in this manuscript:

BMI	body mass index
DIEA	deep inferior epigastric artery
DIEP	deep inferior epigastric perforator
IACUC	Institutional Animal Care and Use Committee
IMA	internal mammary artery
IRB	Institutional Review Board
MS-TRAM	muscle-sparing transverse rectus abdominis myocutaneous
SD	standard deviation
SEA	superior epigastric artery
SHAEP	stacked hemi-abdominal extended perforator
ULAM	Unit for Laboratory Animal Medicine
