# Supplementary material for: Everter-Assisted Arterial Coupling in Abdominal-Based Breast Reconstruction: A Preclinical Study and Retrospective Feasibility Case Series
Source: Cancers (Basel). 2026 Oct 16;18(20):3343. doi: 10.3390/cancers18203343 (PMC13702455; doi:10.3390/cancers18203343)
Supplement: Supplementary file 1 [file cancers-18-03343-s001.zip › cancers-4516194-supplementary.pdf]

Supplementary Materials

Everter-Assisted Arterial Coupling in Abdominal-Based Breast Reconstruction: A Preclinical Study and Retrospective Feasibility Case Series

Table S1. Clinical characteristics, arterial coupling outcomes, and complications.

Patient / breast	Flap	BMI (kg/m²)	Weight (g)	Coupler (mm)	Arterial outcome	Arterial time (min)	Ischemia time (min)	Perioperative and postoperative events and treatment
1R	DIEP	23.6	426	2.0	Success	4.97	42	Early: left breast hematoma (POD1), washout; likely muscle bleeding. Late: right abdominal drain-site cellulitis, antibiotics; left breast fat necrosis, liposuction and fat grafting.
1L	DIEP	23.6	436	2.0	Success	8.57	54	
2R	SHAEP	21.2	318	2.0	Success	18.07	99*	Abdominal wound dehiscence; dressings.
2L	SHAEP	21.2	300	2.0	Success	4.83	122*	
3L	DIEP	24.7	452	2.0	Technical conversion†	NR	94	Small hematoma (POD9), site unspecified; drain retained longer. Intraoperative loss of venous signal; second venous anastomosis.
4R	DIEP	26.4	459	2.0	Success	7.53	41	None recorded.
4L	DIEP	26.4	450	NA	Primary hand-sewn	26.22	87	
5R	DIEP	27.2	549	2.0	Success	9.78	41	Fat necrosis, side unspecified; liposuction and fat grafting.
5L	DIEP	27.2	493	NA	Primary hand-sewn	NR	109	
6R	MS-TRAM	38.7	1004	2.0	Success	11.17	57	Abdominal wound dehiscence; dressings.
6L	DIEP	38.7	999	NA	Primary hand-sewn	20.00	87	
7R	DIEP	46.2	1311	NA	Primary hand-sewn	6.43	48	Right breast wound dehiscence on the primary hand-sewn side; dressings.
7L	MS-TRAM	46.2	1229	2.0	Success	8.57	51	
8R	DIEP	37.6	854	2.0	Thrombotic conversion‡	6.48	73	None recorded.
8L	DIEP	37.6	820	NA	Primary hand-sewn	21.08	43	
9R	DIEP	33.5	1020	NA	Primary hand-sewn	29.72	80	Early: left breast hematoma (POD0), washout; transected lateral perforator. Later: left hematoma (POD6), image-guided drainage; fat necrosis, side not separately specified, excision and fat grafting.
9L	DIEP	33.5	940	2.0	Thrombotic conversion‡	7.33	55	
10R	DIEP	30.4	811	NA	Primary hand-sewn	18.00	76	None recorded.
10L	DIEP	30.4	725	2.0	Thrombotic conversion‡	17.50	85	
11L	DIEP	38.1	1359	2.0	Technical conversion†	6.90§	49	None recorded.

Patient / breast	Flap	BMI (kg/m^2)	Weight (g)	Coupler (mm)	Arterial outcome	Arterial time (min)	Ischemia time (min)	Perioperative and postoperative events and treatment
12R	DIEP	31.2	NR	2.5	Success	NR	70	None recorded.
12L	DIEP	31.2	761	NA	Primary hand-sewn	NR	90	

Notes to Table S1: Each row represents one reconstructed breast. The recipient artery was the internal mammary artery. Coupler size was selected with an intraoperative vessel sizer. Flap weights correspond to the contralateral abdominal donor side. Complications are reported once per patient in merged cells. No flap loss was observed. * Patient 2 contributed two SHAEP flaps, each containing DIEP and SCIP components. Both ischemia times were excluded from analysis because additional intraflap anastomoses were performed after pedicle clipping and before IMA-DIEA anastomosis. Both flaps remain in the cohort and recipient arterial timing analysis; the additional connections were excluded from arterial timing. † In both technical failures, recoil prevented stable eversion and pin engagement; the coupler was not closed and clamps were not released. ‡ In all three thrombotic failures, initial flow was established, then compromised before flap inset. Thrombosis at the coupler was identified, the segment was excised, the lumen was flushed, and a hand-sewn anastomosis was performed without attempted coupler revision. These times measure initial coupling and exclude rescue. § The 6.90-minute technical attempt was abandoned before clamp release; its timing endpoint was not confirmed. BMI, body mass index; DIEP, deep inferior epigastric perforator; IMA-DIEA, internal mammary artery to deep inferior epigastric artery; MS-TRAM, muscle-sparing transverse rectus abdominis myocutaneous; SHAEP, stacked hemi-abdominal extended perforator; SCIP, superficial circumflex iliac artery perforator; POD, postoperative day; NR, not recorded; NA, no arterial coupler attempted.

Table S2. Complete within-patient comparisons of arterial anastomosis and flap ischemia times.

Measure	Patient	Successful coupling (min)	Primary hand-sewn (min)	Hand-sewn minus coupled (min)
Arterial anastomosis time	4	7.53	26.22	18.68
	6	11.17	20.00	8.83
	7	8.57	6.43	-2.13
Flap ischemia time	4	41	87	46
	5	41	109	68
	6	57	87	30
	7	51	48	-3
	12	70	90	20

Positive differences indicate a longer primary hand-sewn time. Differences were calculated before rounding. Arterial comparisons include three complete pairs; ischemia comparisons include five. The two SHAEP flaps were excluded from ischemia analysis and do not appear among these pairs. Clinical timing comparisons are descriptive.

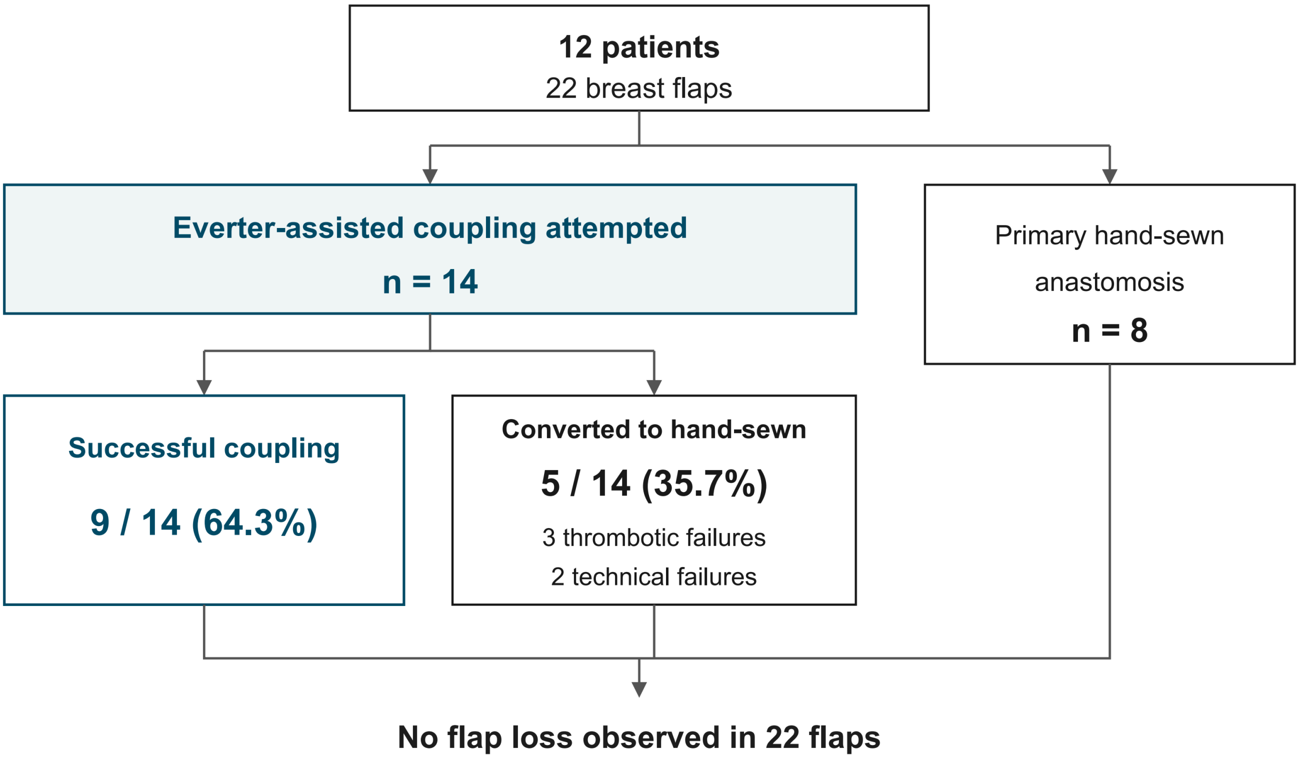

Figure S1. Clinical outcomes of everter-assisted arterial coupling. Arterial measurements were available for 18 flaps: 8 successful couplings, 4 converted attempts and 6 primary hand-sewn anastomoses; the remaining four measurements were unavailable. Converted-attempt measurements excluded rescue time, and one technical attempt was abandoned before clamp release. Ischemia analysis included 20 flaps: 7 successful couplings, 5 converted attempts and 8 primary hand-sewn anastomoses. The two SHAEP flaps were excluded only from ischemia analysis because their ischemic intervals included additional intraflap anastomoses.
